# Prevalence of reduced eGFR in European adults using KDIGO and age-adapted eGFR thresholds

**DOI:** 10.1093/ndt/gfaf112

**Published:** 2025-07-17

**Authors:** Megan E Astley, Nicholas C Chesnaye, Giovanni Gambaro, Alberto Ortiz, Stein Hallan, Juan-Jesus Carrero, Natalie Ebert, Bjørn Odvar Eriksen, Anne-Laure Faucon, Pietro Manuel Ferraro, Till Ittermann, Arnar J Jonsson, Knut Asbjørn Rise Langlo, Toralf Melsom, Elke Schaeffner, Sylvia Stracke, Runolfur Palsson, Kitty J Jager, Vianda S Stel

**Affiliations:** Amsterdam UMC location University of Amsterdam, ERA Registry, Department of Medical Informatics, Amsterdam, The Netherlands; Amsterdam Public Health Research Institute, Health Behaviours and Chronic Diseases and Methodology, Amsterdam, The Netherlands; Amsterdam UMC location University of Amsterdam, ERA Registry, Department of Medical Informatics, Amsterdam, The Netherlands; Amsterdam Public Health Research Institute, Quality of Care, Amsterdam, The Netherlands; Division of Nephrology and Dialysis, Department of Medicine, University of Verona, Verona, Italy; Department of Nephrology and Hypertension, IIS-Fundacion Jimenez Diaz UAM, Madrid, Spain; Department of Clinical and Molecular Medicine, Faculty of Medicine and Health Sciences, Norwegian University of Science and Technology, Trondheim, Norway; Department of Nephrology, St Olavs Hospital, Trondheim, Norway; Department of Medical Epidemiology and Biostatistics, Karolinska Institutet, Stockholm, Sweden; Division of Nephrology, Department of Clinical Sciences, Danderyd Hospital, Danderyd, Sweden; Charité Universitätsmedizin Berlin, Institute of Public Health, Berlin, Germany; Section of Nephrology, Clinic of Internal Medicine, University Hospital of North Norway, Tromsø, Norway; Metabolic and Renal Research Group, UiT The Arctic University of Norway, Tromsø, Norway; Department of Medical Epidemiology and Biostatistics, Karolinska Institutet, Stockholm, Sweden; Division of Nephrology and Dialysis, Department of Medicine, University of Verona, Verona, Italy; Institute for Community Medicine – SHIP Clinical-Epidemiological Research, University Medicine Greifswald, Greifswald, Germany; Internal Medicine Services, Landspitali University Hospital, Reykjavik, Iceland; Faculty of Medicine, School of Health Sciences, University of Iceland, Reykjavik, Iceland; Department of Clinical and Molecular Medicine, Faculty of Medicine and Health Sciences, Norwegian University of Science and Technology, Trondheim, Norway; Department of Nephrology, St Olavs Hospital, Trondheim, Norway; Section of Nephrology, Clinic of Internal Medicine, University Hospital of North Norway, Tromsø, Norway; Metabolic and Renal Research Group, UiT The Arctic University of Norway, Tromsø, Norway; Charité Universitätsmedizin Berlin, Institute of Public Health, Berlin, Germany; Department of Internal Medicine A, Section of Nephrology, University Medicine Greifswald, Greifswald, Germany; Faculty of Medicine, School of Health Sciences, University of Iceland, Reykjavik, Iceland; Section of Nephrology, Landspitali University Hospital, Reykjavik, Iceland; Amsterdam UMC location University of Amsterdam, ERA Registry, Department of Medical Informatics, Amsterdam, The Netherlands; Amsterdam Public Health Research Institute, Quality of Care, Amsterdam, The Netherlands; Amsterdam UMC location University of Amsterdam, ERA Registry, Department of Medical Informatics, Amsterdam, The Netherlands; Amsterdam Public Health Research Institute, Quality of Care, Amsterdam, The Netherlands

**Keywords:** age-adapted, chronic kidney disease, eGFR, KDIGO, prevalence

## Abstract

**Background:**

The current definition for chronic kidney disease (CKD) does not account for age-related kidney function decline. Age-adapted CKD definitions have been suggested, but their impact on the estimates of reduced kidney function prevalence remains unclear. We aimed to compare the prevalence of reduced estimated glomerular filtration rate (eGFR) in European adults using the KDIGO eGFR threshold and three age-adapted eGFR thresholds.

**Methods:**

This cross-sectional study included data collected after 1999 from nine general population-based studies from seven European countries. eGFR was calculated using the European Kidney Function Consortium equation. Prevalence of reduced eGFR was estimated over age using four eGFR thresholds: the KDIGO eGFR threshold (eGFR <60 mL/min/1.73 m^2^), the categorical age-adapted eGFR threshold (eGFR <75 mL/min/1.73 m^2^ if aged <40 years, eGFR <60 mL/min/1.73 m^2^ if aged 40–65 years, eGFR <45 mL/min/1.73 m^2^ if aged >65 years), and two novel continuous age- and sex-adapted eGFR thresholds based on the 5th and 2.5th percentile of eGFR in healthy individuals.

**Results:**

We used data from over 2.5 million European adults (46% men). Among those under 40 years, the KDIGO threshold provided the lowest average prevalence estimate (<1%), followed by the categorical age-adapted threshold (3%) and the 2.5th and 5th continuous age- and sex-adapted thresholds (4% and 7%). In adults over 65 years, the 2.5th continuous age- and sex-adapted threshold (10%) estimated the lowest prevalence, followed by the 5th continuous age- and sex-adapted threshold (15%), categorical age-adapted threshold (19%) and KDIGO threshold (45%).

**Conclusion:**

Using age-adapted eGFR thresholds would likely cause a shift in the prevalence of reduced kidney function. However, the association between these novel age-adapted thresholds and clinical outcomes needs to be established before considering their incorporation into the definition of CKD.

## Graphical Abstract

**Graphical Abstract:**
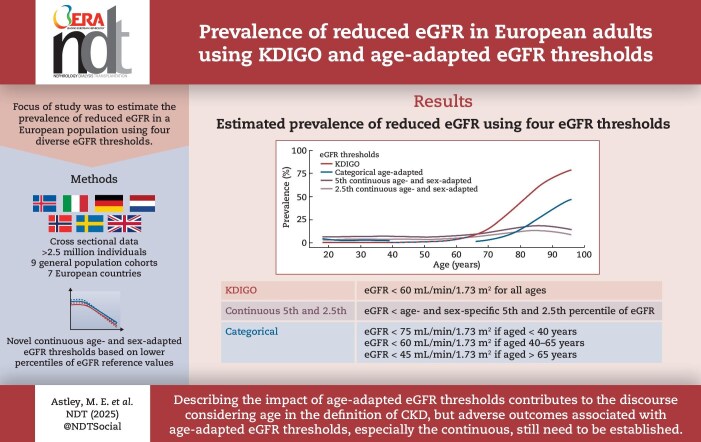

KEY LEARNING POINTS **What was known:**

- Kidney function decreases with age, even in healthy individuals with no other signs of kidney damage or disease.
- To account for this age-related decrease in kidney function, age-adapted estimated glomerular filtration rate (eGFR) thresholds to define chronic kidney disease (CKD) have been suggested.
- A limited number of studies have reported the prevalence of reduced eGFR using a categorical age-adapted CKD definition and none have presented or used a continuous age-adapted eGFR threshold.

**This study adds:**

- We present the first continuous age- and sex-adapted eGFR thresholds based on the lower 5th and 2.5th percentile of eGFR references values derived from over 1.5 million healthy individuals.
- This is the first study to estimate the prevalence of reduced eGFR using the KDIGO eGFR threshold, categorical age-adapted eGFR threshold and two continuous age-adapted eGFR thresholds to define reduced eGFR over a full age range, stratified by sex, in a multinational European cohort.
- Compared with the KDIGO eGFR threshold, our results show a higher prevalence of reduced eGFR in younger adults and lower prevalence in older adults when applying age-adapted eGFR thresholds.

**Potential impact:**

- This study highlights the effect of age-adapted eGFR thresholds on prevalence of reduced eGFR, contributing to the discourse regarding the incorporation of age in the definition of CKD.
- The association of clinical outcomes with age-adapted eGFR thresholds needs to be established to determine their value in risk stratification and management of CKD.

## INTRODUCTION

Chronic kidney disease (CKD) is often diagnosed when the estimated glomerular filtration rate (eGFR) is below 60 mL/min/1.73 m² or the urine albumin-to-creatinine ratio (UACR) is 30 mg/g or higher, based on KDIGO guidelines [1]. However, the KDIGO CKD definition has been questioned as it does not account for age-related changes in kidney function and what may be considered abnormal or normal eGFR depends on an individual's age [2–8]. A majority of adults aged below 45 years with CKD are diagnosed based on elevated UACR without having a concurrent eGFR below the KDIGO eGFR threshold [9]. However, UACR is often not assessed in most individuals in primary care settings [10, 11], and as a result, some younger adults with reduced kidney function may not be identified using the KDIGO CKD definition if only eGFR is calculated. Conversely, an eGFR below 60 mL/min/1.73 m^2^ may not be abnormal for older adults who experienced age-related lowering of eGFR. Additionally, studies have shown the risk of adverse outcomes increases at eGFR levels higher than 60 mL/min/1.73 m^2^ in younger adults [3, 12, 13] and below 60 mL/min/1.73 m^2^ in older adults [3, 14]. These considerations have led to discourse regarding the incorporation of age-related eGFR decline in the definition of CKD to identify adults with abnormally low kidney function [5, 8, 15].

To account for kidney ageing, some authors have advocated using an age-adapted definition of CKD [3, 4, 14], for example, by using three age groups with progressively lower eGFR thresholds such as eGFR <75 mL/min/1.73 m^2^ for those aged below 40 years, eGFR <60 mL/min/1.73 m^2^ for those aged 40–65 years and eGFR <45 mL/min/1.73 m^2^ for those aged above 65 years [3]. However, there are biological and logical concerns about reclassifying individuals from having CKD to not having CKD when they transition into the next age group without any change in their eGFR. An alternative approach involves using a continuous age-adapted eGFR threshold based on a lower percentile of eGFR reference values derived from a healthy population [3, 16].

Only a few studies have reported the shift in estimated prevalence of CKD when using the categorical age-adapted CKD definition compared with the standard KDIGO CKD definition [14, 17, 18]. However, to our knowledge, no previous study has described or used a continuous age- and sex-adapted eGFR threshold to estimate the prevalence of reduced eGFR. Further research into age-adapted eGFR thresholds is warranted to obtain insights which can inform discussions regarding the consideration of age in the definition of CKD.

We therefore aimed to compare the prevalence of reduced eGFR over age in more than 2.5 million adults from a multinational European general population using the KDIGO eGFR threshold, the categorical age-adapted eGFR threshold, and two novel continuous age- and sex-adapted eGFR thresholds based on the 5th and 2.5th percentile of eGFR in healthy adults.

## MATERIALS AND METHODS

### Selection of participating studies

For this cross-sectional study, general population studies from Europe were identified for inclusion in the European CKD Burden Consortium by a literature database search, outreach, and from a previous study of the European CKD Burden Consortium [19]. Studies were included if they were designed to select a representative sample of the target general population, had a minimum sample size of 2000 individuals, and included data after 1999 from adults aged 18 years and older. Description of recruitment procedures for participating studies is described elsewhere [20].

### Data collection and determination of eGFR

Data collection dates varied among individuals within each study and ranged from January 2000 to April 2024. Serum creatinine was determined by Jaffe or enzymatic assays and traceable to isotope dilution mass spectrometry (IDMS). The creatinine-based eGFR was calculated using the European Kidney Function Consortium (EKFC) equation with sex-specific Q values of 0.9 mg/dL in men and 0.7 mg/dL in women older than 25 years [21]. In those under 25 years, Q values were calculated using an age- and sex-specific equation [21]. We repeated the analyses using the 2009 Chronic Kidney Disease Epidemiology Collaboration (CKD-EPI_2009_) [22] and the CKD-EPI_2021_ equation [23]. CKD-EPI_2009_ was used in a race-agnostic manner and race was considered non-Black for all participants in the calculation of eGFR as most studies comprised a predominantly White study population and did not collect data on race, or were not allowed to collect such information.

### Reduced eGFR thresholds

**Figure 1:**
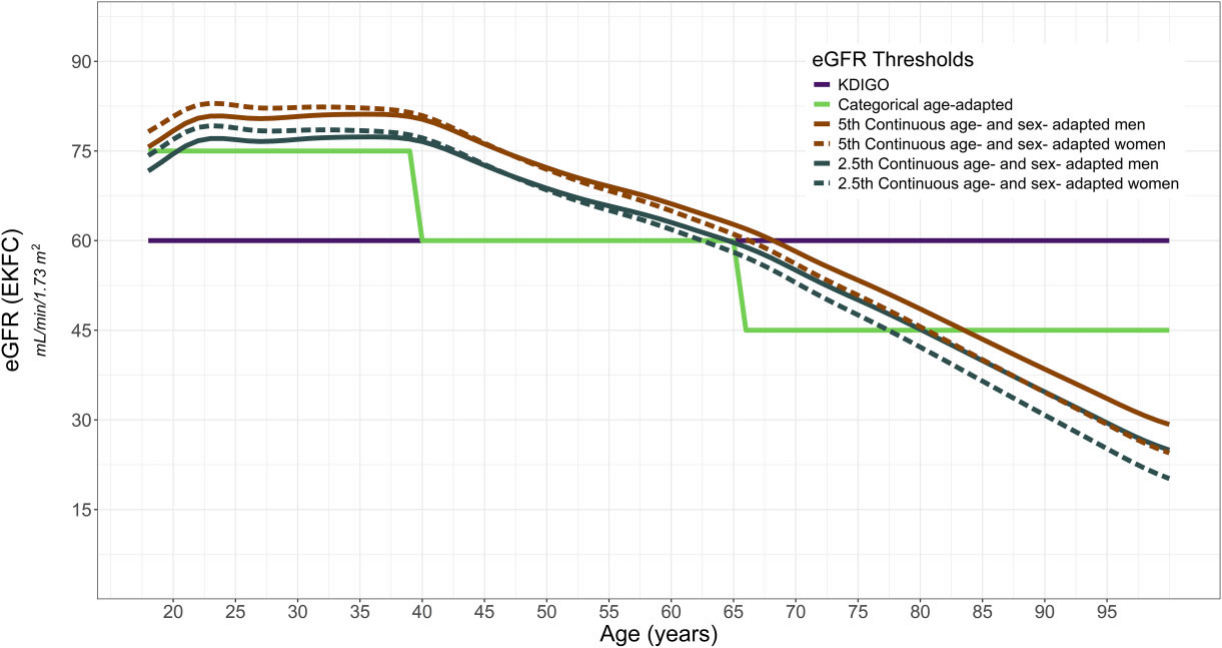
Four eGFR thresholds used to identify individuals with reduced eGFR based on eGFR calculated by the EKFC equation. The eGFR thresholds presented are the KDIGO eGFR threshold [1], the categorical age-adapted eGFR threshold [3], the 5th continuous age- and sex-adapted eGFR, and the 2.5th continuous age- and sex-adapted eGFR threshold; the latter two are presented in men and women separately. For sex-specific thresholds, the colour is used to represent the threshold and line type is used to represent sex (dashed line for women, solid line for men).

Four eGFR thresholds were used to identify individuals with reduced eGFR (Fig. 1), according to eGFR calculated using the EKFC equation. First, the KDIGO eGFR threshold from the KDIGO definition of CKD categories G3 to G5 which requires an eGFR <60 mL/min/1.73 m^2^ for three consecutive months [1]. Because only baseline serum creatinine was available, the chronicity criterion of the KDIGO CKD definition was not applied to identify those with reduced eGFR. Second, the categorical age-adapted eGFR threshold which used three age groups with specific eGFR thresholds: <75 mL/min/1.73 m² for individuals under age 40 years old, <60 mL/min/1.73 m² for those aged 40–65 years old and <45 mL/min/1.73 m² for those over 65 years old [3]. Third, the 5th continuous age- and sex-adapted eGFR threshold derived from the 5th percentile of eGFR distribution in a healthy adult (≥18 years) population, based on a previous European CKD Burden Consortium study [20]. Fourth, the 2.5th continuous age- and sex-adapted eGFR threshold derived from the 2.5th percentile of eGFR distribution [20].

### Statistical analysis

**Figure 2:**
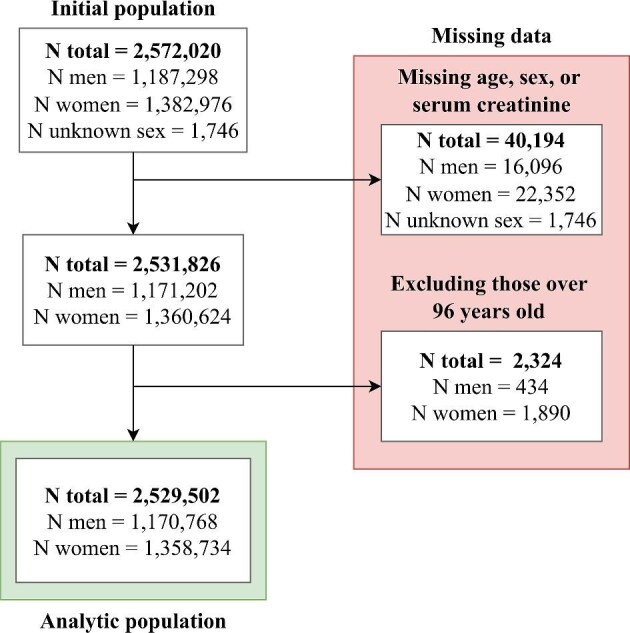
Flowchart showing the inclusion process for individuals in the study population. Individuals with missing data on age, sex or serum creatinine were excluded. Those older than 96 years were excluded to align with the maximum age of individuals in the development cohort of the EKFC equation [21].

Normally distributed continuous variables were presented as means with standard deviation (SD). Skewed continuous variables were presented as medians with interquartile range (IQR). Categorical variables were presented as frequencies with percentages. Individuals missing data on age, sex or serum creatinine were not included in the analysis. Individuals older than 96 years were also excluded from the analysis as this is the maximum age of individuals included in the development dataset for the EKFC equation (Fig. 2) [21]. Age- and sex-specific prevalence of reduced eGFR is presented with 95% confidence intervals. Prevalence was modelled smoothly over age with a generalized additive model using the ‘mgcv’ R package [24]. Prevalence was also modelled per cohort over the age range of the cohort. Results obtained using the CKD-EPI_2009_ and CKD-EPI_2021_ equations, overall and also by sex, are presented in the Supplementary data. All analyses were carried out in R version 4.4.1 [25].

## RESULTS

### Characteristics of the pooled study cohort

We included nine population-based studies which were from Germany [the Berlin Initiative Study (BIS) and Study of Health in Pomerania (SHIP)], Iceland (the Iceland CKD Study), Italy [Initiative on Nephropathy, of relevance to public health, which is Chronic, possibly in its Initial stages, and carries a Potential risk of major clinical Endpoints (INCIPE)], the Netherlands [Lifelines Cohort Study (Lifelines)], Norway [the Nord-Trøndelag Health Study (HUNT) Study and the Tromsø Study], Sweden Stockholm CREAtinine Measurements (SCREAM) and the UK (the UK Biobank) (Table 1) [20]. Most individuals came from SCREAM (64%), the UK Biobank (19%), the Iceland CKD Study (9%), Lifelines (6%) and the HUNT Study (2%), while the remaining studies contributed less than 1% to the analytic cohort. Six studies covered a relatively full age range, whereas the remaining studies did not include individuals under the age of 40 years. The median age distribution in these studies ranged from 45 to 79 years. The proportion of men in studies ranged from 42% to 48%.

**Table 1:** Description of participating studies and sampling characteristics from the European CKD Burden Consortium.

									
Country	Region(s) or cities	Study	*N* individuals (% men)	Age range, years^a^	Median age in years (IQR)	Sampling frame	Serum creatinine determination	Sample selection	Response (%)
									
Iceland	All	Iceland CKD Study [17]	218 275 (47%)	18+	46 (32–60)	National health service	Enzymatic method	Inhabitants of Iceland with 1 or more serum creatinine measurements available	66
Italy	Northeast	INCIPE [52]	3867 (48%)	40+	59 (50–68)	General practitioners lists	Jaffe method	Random selection of participants from 62 random selected practices	62
Germany	Berlin	BIS [53]	2061 (47%)	70+	79 (74–85)	Health insurance registers	Enzymatic method	Random selection of participants insured by the AOK Nordost	8.1
	Northeast	SHIP [54]	4413 (48%)	20–77	53 (40–64)	Population registers	Jaffe method	Stratification based on number of residents per municipality. Age- and sex- stratified random sample selection per community	69
Netherlands	Northern	Lifelines [55]	145 177 (42%)	18+	45 (36–52)	General practitioners lists	Enzymatic method	General practitioners invited patients to participate, and patients invited family members to participate	58–84^b^
Norway	Central	The HUNT Study [56]	49 961 (45%)	20+	54 (41–64)	Census data	Jaffe method	All residents in region	54
	Tromsø municipality	The Tromsø Study [57]	20 973 (48%)	40+	56 (48–66)	Health registries	Enzymatic method	Random selection of individuals from birth cohorts and population registries were invited to participate	65
Sweden	Stockholm	SCREAM [58]	1 614 461 (47%)	18+	47 (33–63)	Health and laboratory registries	Jaffe method and enzymatic method	All Stockholm residents with a valid personal identifying number who had at least one measurement of creatinine available	69^c^
UK	England, Wales and Scotland	UK Biobank [59]	470 314 (46%)	40–70	58 (50–63)	General practitioners lists	Enzymatic method	All individuals on National Health Service patient registers living within a reasonable travelling distance of an assessment centre	5.5

a Studies with data on all ages were asked to provide only data on those aged 18 years and above.

b Dependent on questionnaire.

c This is the proportion of the sampling population that underwent serum creatinine testing during the study period.

### Population characteristics by sex

There were 2 572 020 participants, 2 531 826 (98%) of whom had complete data available (Fig. 2). Another 2324 individuals (0.09%) older than 96 years were excluded from the analysis, yielding a final study population of 2 529 502. The population characteristics are presented by sex in Table 2. Forty-six percent of the participants were men. The median age was 50 years for both men and in women. The median eGFR was 89 mL/min/1.73 m^2^ for both men and women.

**Table 2:** Population characteristics of individuals aged 18–96 years old by sex for each study.

										
	Iceland CKD Study (Iceland)	INCIPE (Italy)	BIS (Germany)	SHIP (Germany)	Lifelines (Netherlands)	The HUNT Study (Norway)	The Tromsø Study (Norway)	SCREAM (Sweden)	UK Biobank (The UK)	Total
										
Men										
Number of men (% of cohort)	102 456 (47%)	1846 (48%)	977 (47%)	2140 (48%)	60 446 (42%)	22 692 (45%)	9964 (48%)	754 885 (47%)	215 362 (46%)	1 170 768 (46%)
Age range in years, min–max	18–96	26–95	70–96	21–84	18–92	19–96	40–95	18–96	37–73	18–96
Age in years, mean (SD)	48 (18)	60 (11)	80 (7)	53 (16)	45 (13)	54 (16)	57 (11)	48 (18)	57 (8)	50 (17)
Mean eGFR by EKFC equation^a^ in mL/min/1.73 m^2^, mean (SD)	87 (19)	82 (16)	58 (16)	85 (19)	94 (14)	91 (18)	85 (15)	90 (18)	87 (13)	89 (17)
Mean eGFR by CKD-EPI_2009_ in mL/min/1.73 m^2^, mean (SD)	92 (21)	86 (16)	65 (18)	89 (19)	97 (16)	96 (18)	89 (15)	95 (20)	90 (13)	94 (19)
Mean eGFR by CKD-EPI_2021_ in mL/min/1.73 m^2^, mean (SD)	96 (20)	90 (16)	69 (19)	92 (19)	101 (15)	100 (17)	93 (14)	99 (19)	95 (13)	98 (18)
Women										
Number of women (% of cohort)	115 819 (53%)	2021 (52%)	1084 (53%)	2273 (52%)	84 731 (58%)	27 269 (55%)	11 009 (52%)	859 576 (53%)	254 952 (54%)	1 358 734 (54%)
Age range in years, min–max	18–96	24–93	69–96	20–83	18–93	19–96	40–96	18–96	39–71	18–96
Age in years, mean (SD)	46 (19)	60 (12)	79 (7)	51 (15)	44 (13)	53 (16)	57 (11)	48 (19)	56 (8)	50 (18)
Mean eGFR by EKFC equation^a^ in mL/min/1.73 m^2^, mean (SD)	87 (20)	80 (17)	58 (15)	83 (19)	91 (14)	89 (19)	84 (15)	90 (20)	86 (13)	89 (18)
Mean eGFR by CKD-EPI_2009_ equation in mL/min/1.73 m^2^, mean (SD)	92 (23)	84 (17)	64 (17)	87 (20)	96 (16)	94 (20)	88 (15)	96 (22)	90 (14)	94 (20)
Mean eGFR by CKD-EPI_2021_ equation in mL/min/1.73 m^2^, mean (SD)	96 (22)	89 (16)	69 (18)	91 (20)	100 (16)	98 (19)	92 (15)	100 (21)	95 (13)	98 (19)

Individuals with missing data are excluded from this table.

a EKFC equation.

### Prevalence of reduced eGFR across the age range

**Figure 3:**
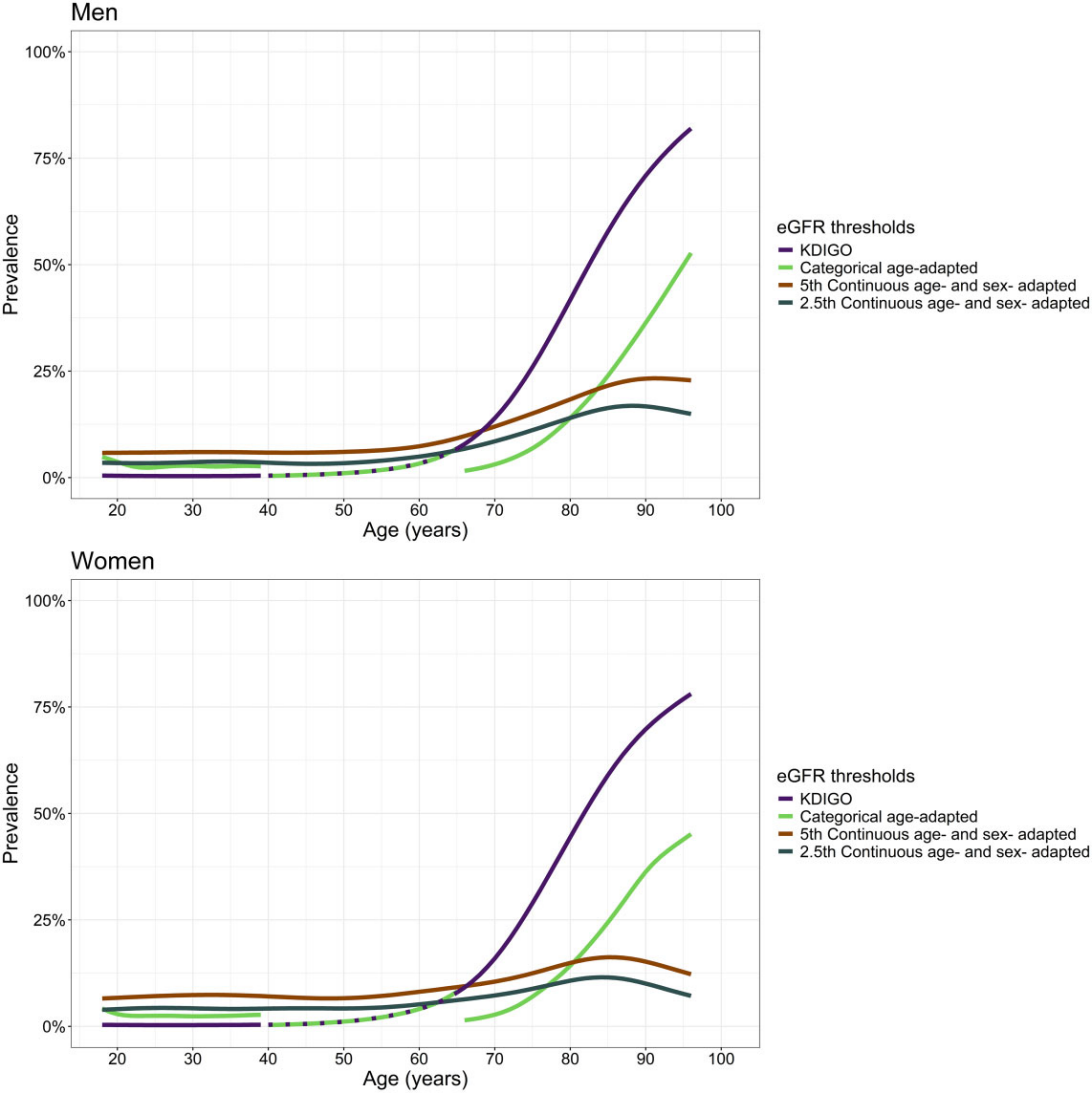
Prevalence of reduced eGFR in individuals aged 18–96 years using four eGFR thresholds (**A**) to define reduced eGFR, stratified by sex. eGFR was calculated using the EKFC equation (**B**). Because the KDIGO and categorical age-adapted eGFR thresholds are the same from the ages of 40–65 years, the lines for this age range are overlapping and this is represented by the dashed purple line overlapping the solid green line. (A) The four thresholds are: the KDIGO eGFR threshold (eGFR <60 mL/min/1.73 m^2^), the categorical age-adapted eGFR threshold (eGFR <75 mL/min/1.73 m^2^ for those aged below 40 years, eGFR <60 mL/min/1.73 m^2^ for those aged 40–65 years, eGFR <45 mL/min/1.73 m^2^ for those aged above 65 years), the 5th continuous age- and sex-adapted eGFR threshold (5th percentile of eGFR from a healthy population), and the 2.5th continuous age- and sex-adapted eGFR threshold (2.5th percentile of eGFR from a healthy population). (B) The EKFC equation.

Estimated prevalence of reduced eGFR for men and women using the KDIGO, categorical age-adapted, 5th continuous age- and sex-adapted, and 2.5th continuous age- and sex-adapted eGFR thresholds are shown in Fig. 3 and Table 3. Results not stratified by sex are presented in Supplementary data, Table S2 and Fig. S1 and for each study in Supplementary data, Fig. S2. Results using the CKD-EPI_2009_ and CKD-EPI_2021_ equations are presented in Supplementary data, Tables S3 and S4, and Figs S3 and S4.

**Table 3:** Estimated prevalence of reduced eGFR presented per year of age by 5-year age intervals and for the oldest possible year of age (96 years), stratified by sex.

Estimated prevalence of reduced eGFR using four eGFR thresholds, % (95% CI)				
Age in years	KDIGO	Categorical	5th continuous	2.5th continuous
Men				
20	0.4 (0.4–0.5)	3.7 (3.5–3.9)	5.8 (5.7–6.0)	3.4 (3.3–3.6)
25	0.4 (0.3–0.4)	2.4 (2.2–2.5)	5.9 (5.8–6.0)	3.4 (3.3–3.5)
30	0.3 (0.3–0.4)	2.8 (2.6–2.9)	6.0 (5.9–6.1)	3.6 (3.5–3.7)
35	0.4 (0.3–0.4)	2.7 (2.5–2.8)	6.0 (5.8–6.1)	3.7 (3.6–3.8)
40	0.4 (0.4–0.5)	0.4 (0.3–0.4)	5.9 (5.8–6.0)	3.5 (3.4–3.6)
45	0.6 (0.6–0.7)	0.6 (0.6–0.7)	5.9 (5.8–6.0)	3.2 (3.2–3.3)
50	1.0 (1.0–1.0)	1.0 (1.0–1.1)	6.0 (5.9–6.1)	3.4 (3.3–3.5)
55	1.8 (1.7–1.8)	1.8 (1.7–1.8)	6.4 (6.3–6.5)	4.0 (3.9–4.0)
60	3.3 (3.3–3.4)	3.3 (3.2–3.4)	7.4 (7.2–7.5)	4.9 (4.8–5.0)
65	6.8 (6.7–6.9)	6.9 (6.6–7.1)	9.2 (9.1–9.4)	6.4 (6.3–6.5)
70	13.9 (13.7–14.1)	3.1 (3.0–3.2)	12 (11.8–12.2)	8.5 (8.4–8.7)
75	26.0 (25.6–26.3)	6.9 (6.7–7.1)	15.1 (14.8–15.3)	11.1 (10.9–11.4)
80	41.7 (41.3–42.2)	14.0 (13.6–14.4)	18.4 (18.0–18.7)	14.0 (13.7–14.3)
85	57.8 (57.2–58.4)	23.9 (23.3–24.5)	21.6 (21.1–22.1)	16.3 (15.9–16.8)
Women				
20	0.3 (0.3–0.4)	2.9 (2.7–3)	6.7 (6.6–6.9)	4 (3.9–4.2)
25	0.3 (0.3–0.3)	2.5 (2.4–2.6)	7.1 (7.0–7.2)	4.3 (4.2–4.4)
30	0.3 (0.3–0.3)	2.4 (2.2–2.5)	7.3 (7.2–7.5)	4.2 (4.1–4.3)
35	0.3 (0.3–0.3)	2.5 (2.4–2.6)	7.3 (7.2–7.5)	4.1 (4.0–4.2)
40	0.4 (0.4–0.4)	0.3 (0.3–0.3)	7.0 (6.9–7.1)	4.2 (4.1–4.3)
45	0.6 (0.5–0.6)	0.6 (0.6–0.6)	6.7 (6.6–6.8)	4.2 (4.2–4.3)
50	1.1 (1.0–1.1)	1.1 (1.1–1.2)	6.6 (6.5–6.7)	4.2 (4.1–4.3)
55	2.2 (2.1–2.2)	2.1 (2.0–2.2)	7.1 (7.0–7.2)	4.4 (4.3–4.5)
60	4.1 (4.0–4.2)	4.1 (4.0–4.2)	8.1 (8.0–8.2)	5.2 (5.1–5.3)
65	7.9 (7.8–8.1)	8.2 (7.9–8.4)	9.2 (9.1–9.3)	6.2 (6.1–6.3)
70	15.9 (15.7–16.2)	2.7 (2.6–2.9)	10.5 (10.3–10.7)	7.3 (7.1–7.4)
75	28.9 (28.6–29.3)	7.0 (6.8–7.2)	12.5 (12.2–12.7)	8.8 (8.6–9.0)
80	44.4 (44–44.8)	14.2 (13.9–14.6)	14.9 (14.6–15.2)	10.7 (10.5–11)
85	59.0 (58.5–59.5)	24.4 (23.9–25.0)	16.2 (15.9–16.6)	11.5 (11.2–11.8)

Prevalence was estimated using four eGFR thresholds abbreviated as follows: KDIGO = KDIGO eGFR threshold; Categorical = categorical age-adapted eGFR threshold; 5th continuous = 5th continuous age- and sex-adapted eGFR threshold; 2.5th continuous = 2.5th continuous age- and sex-adapted eGFR threshold.

All values are rounded to one decimal point.

Results are presented as a percentage with 95% confidence intervals (95% CIs).

### Prevalence of reduced eGFR in individuals aged under 40 years

In men aged 18 years, the prevalence of reduced eGFR estimated using the KDIGO, categorical age-adapted, 5th continuous age- and sex-adapted, and 2.5th continuous age- and sex-adapted eGFR thresholds were 0.5%, 5%, 6% and 3.5%, respectively, and remained stable until the age of 39 years.

In women aged 18 years, the prevalence of reduced eGFR estimated using KDIGO, categorical age-adapted, 5th continuous age- and sex-adapted, and 2.5th continuous age- and sex-adapted eGFR thresholds were 0.3%, 4%, 7% and 4%, respectively. This also stayed relatively stable until the age of 39 years.

### Prevalence of reduced eGFR in individuals aged 40–65 years

Since the KDIGO eGFR threshold and the age-adapted categorical eGFR threshold are the same for those aged 40–65 years, we will not present results using the latter.

In men aged 40 years, reduced eGFR prevalence estimated using the KDIGO, 5th continuous age- and sex-adapted, and 2.5th continuous age- and sex-adapted eGFR thresholds were 0.4%, 6% and 4%, respectively. By age 65 years, prevalence increased to 7%, 9% and 6%, respectively.

In women aged 40 years, reduced eGFR prevalence estimated using the KDIGO, 5th continuous age- and sex-adapted, and 2.5th continuous age- and sex-adapted eGFR thresholds were 0.3%, 7% and 4%, respectively. By the age of 65 years, prevalence increased to 8%, 9% and 6%, respectively.

### Prevalence of reduced eGFR in individuals aged 66–96 years

As the previous age groupings spanned over about 20 years of age, we similarly present prevalence for a roughly 20 years age span. Reduced eGFR prevalence estimated using the KDIGO, categorical age-adapted, 5th continuous age- and sex-adapted, and 2.5th continuous age- and sex-adapted eGFR thresholds in men aged 66 years was 8%, 2%, 10% and 7%, respectively. By age 85 years, prevalence in men increased to 58%, 24%, 22% and 16%, respectively.

Women had a similar estimated prevalence as men at the age of 66 years using the four eGFR thresholds (9%, 1%, 9% and 6%). By age 85 years, prevalence in women estimated using the KDIGO, categorical age-adapted, 5th continuous age- and sex-adapted, and 2.5th continuous age- and sex-adapted eGFR thresholds were 59%, 24%, 16% and 12%, respectively.

## DISCUSSION

### Summary of findings

The findings of this study demonstrate substantial age-related variations in the prevalence of reduced eGFR when applying the KDIGO, the categorical age-adapted, and both continuous age- and sex-adapted eGFR thresholds. The KDIGO eGFR threshold provided the largest variation in prevalence estimates across age. In contrast, the 5th and 2.5th continuous age- and sex-adapted eGFR thresholds demonstrated less variation in reduced eGFR prevalence across the age span. Prevalence of reduced eGFR, regardless of the threshold used, was not markedly different between men and women aged under 65 years. However, slight sex-based differences in prevalence emerged in older adults and varied according to eGFR threshold.

### Prevalence of reduced eGFR in younger adults

In adults younger than 40 years, the age-adapted eGFR thresholds yielded a higher estimated prevalence of reduced eGFR compared with the KDIGO eGFR threshold, a finding also reported by other studies [17, 26]. The current KDIGO CKD definition has been criticized for not accounting for age, as recent studies have shown ‘normal’ eGFR and eGFR-related risks of adverse outcomes to differ by age [3, 4, 12]. Younger individuals who have abnormally low kidney function for their age [6, 7, 27], while not meeting the KDIGO eGFR threshold, may experience an increased risk of adverse health outcomes, even starting at mild reductions of eGFR such as below 90 mL/min/1.73 m^2^ [3, 12, 13, 28, 29]. Hussain *et al*. showed that individuals aged 18–65 years with an eGFR at or below their age-specific 10th percentile (still exceeding 60 mL/min/1.73 m^2^) had increased risks of mortality, cardiovascular events and kidney failure when compared with individuals with an age-specific median eGFR [12]. Notably, these increased risks were most pronounced in the youngest adults.

Younger adults with reduced eGFR for their age may benefit from identification of reduced eGFR before reaching 60 mL/min/1.73 m^2^, so that interventions aimed at halting or slowing kidney function decline can be initiated at an early stage of the disease and potentially reduce the risk of adverse outcomes associated with reduced eGFR. Such measures include investigations to identify the aetiology of kidney disease and controlling cause-specific risk-factors [30], such as diabetes [31], or prescribing nephroprotective medications, including renin–angiotensin system inhibitors, sodium-glucose cotransporter 2 (SGLT2) inhibitors, or glucagon-like peptide-1 (GLP1) receptor agonists [32–35]. Kushner *et al*. estimated that initiating SGLT2 inhibitor treatment at an eGFR of 75 mL/min/1.73 m^2^ (versus no initiation) could delay the onset of kidney failure by 15 years [36]. However, waiting to initiate treatment until the eGFR reached 60 mL/min/1.73 m^2^ reduced this delay in onset of kidney failure to 11 years [36]. Therefore, it may be advantageous to recognize reduced kidney function in younger adults that is associated with increased risks of adverse outcomes before significant and irreversible reductions in kidney function have occurred.

As the majority of young CKD patients will have increased albuminuria without eGFR <60 mL/min/1.73 m^2^, relying solely on eGFR for a CKD diagnosis might miss younger adults with kidney damage [9]. This is important to consider in primary care settings as only about 30% of individuals have assessment of proteinuria performed, whereas eGFR is determined in over roughly 85% of individuals [10, 11]. However, in a primary care setting, general practitioners may not recognize that age is an important factor to consider in the interpretation of eGFR in younger adults. One study found that general practitioners were more likely to identify a clinical kidney problem in a younger adult with reduced eGFR, which was not yet below 60 mL/min/1.73 m^2^, when presented with age-specific reference values (79% versus 52% of general practitioners) [37]. Many uncertainties remain regarding the relationship between eGFR and age in younger adults, warranting further research on kidney function, related outcomes and management of kidney disease in this age group.

### Prevalence of reduced eGFR in older adults

The use of the KDIGO eGFR threshold in older adults has been questioned as it does not account for the age-related decline in kidney function [5, 8, 15]. Therefore, older adults may be diagnosed with CKD despite having eGFR within the normal reference range for their age, lacking risk factors or other indicators for CKD and being unlikely to experience increased risks of adverse events associated with their eGFR [3, 4, 9, 28, 38]. Additionally, the proportion of older adults UACR criteria for a CKD diagnosis decreases with age, whereas a majority of older individuals with CKD are identified based on the eGFR criteria [9]. This may result in unnecessary referrals to nephrologists and contribute to overburdened healthcare systems, excessive clinical costs and unnecessary patient stress [9]. Nonetheless, older individuals may still benefit from a diagnosis of CKD at an eGFR of 60 mL/min/1.73 m^2^, if they have albuminuria or rapid loss of eGFR, as treatment initiation may slow the progression of kidney function decline [39] and potentially alleviate adverse health outcomes associated with reduced kidney function [38, 40, 41]. Also, adults aged over 75 years may be more likely to experience late referral to a nephrologist, and lowering the eGFR threshold required for a CKD diagnosis may exacerbate this delay [42]. Alternatively, a lower eGFR threshold in older adults could lead to better optimized nephrological resources [43], but this is at the risk of missing those with rapid kidney function loss still with an eGFR above 45 mL/min/1.73 m^2^.

When using the categorical age-adapted definition, CKD prevalence in those older than 65 years old has been reported as 43%–59% lower when compared with prevalence estimates obtained using the KDIGO CKD definition [17, 26]. However, our results illustrate a significant limitation of using a stepwise eGFR threshold; as the prevalence of reduced eGFR in individuals aged 65 years was markedly higher than in individuals aged 66 years. This ‘birthday paradox’ creates a potential problem with identifying reduced eGFR in individuals near margins of an age group. Our approach of using a continuous age- and sex-adapted eGFR threshold avoids this pitfall, showing a continuity of prevalence across the age range.

### Sex differences in the prevalence of reduced kidney function by definition

Although women have a higher prevalence of CKD categories G3 to G4 than men [17, 26], a faster decline in kidney function occurs in men [15, 44], resulting in a higher rate of kidney replacement therapy [45, 46]. However, when using the continuous age- and sex-adapted eGFR threshold, men had a higher prevalence of reduced eGFR than women above the age of 59 years, and the difference became larger with increasing age. This is likely attributable to the sex-specific levels of the continuous age- and sex-adapted eGFR threshold, which are based on sex-specific eGFR reference values. A German study conducted in the general population found that mean eGFR values were not different between men and women, except in healthy individuals where eGFR was slightly lower in women than in men [5]. This difference is probably not caused by the GFR estimating equations, as healthy women from the general population and individuals who have donated a kidney are reported to have lower measured GFR than healthy men [15, 27]. However, when sex-specific thresholds derived from healthy individuals are applied to the general population, women may require a larger decrease in eGFR to reach the sex-specific threshold for reduced kidney function. This highlights the need to investigate the reasons for differences in estimated and measured GFR between men and women, as such reasons may need to be considered when defining kidney disease.

### Strengths and limitations

A significant strength of this study is the estimation of the prevalence of reduced eGFR across multiple large European general population studies the comparison of the KDIGO, categorical age-adapted and two continuous age- and sex-adapted eGFR thresholds, stratified by sex and across a full age range. Additionally, the continuous age- and sex-adapted eGFR thresholds are based on reference values derived from a strict selection of over 1.5 million healthy individuals according to 14 criteria from the same cohort used in our previous analysis [20]. Nevertheless, the generalizability of these results in Eastern Europe (as no studies from this region participated) and outside Europe should be considered with caution.

However, our study is also subject to some limitations. Reduced eGFR was only based on a single determination of eGFR, which results in prevalence estimates higher than when definitions including the 90-day chronicity criterion are used [17]. In addition, our results may be subject to selection bias since a majority of individuals in our cohort were selected because they had at least one available measurement of serum creatinine, suggesting a potential overrepresentation of those with suspected kidney disorder [47]. On the other hand, volunteer-based selection, such as in the case of the UK Biobank, may result in recruitment of individuals in better health than the population they are selected from, potentially leading to an underrepresentation of those with suspected kidney disease or reduced eGFR [48]. Also, eGFR derived from serum creatinine may be biased by non-GFR factors, especially in the older age groups [49, 50]. Unfortunately, cystatin C values, which would have improved the performance of the EKFC equation, were not available [51]. We also lacked data on measured GFR. However, the serum creatinine values from all studies were IDMS-standardized to reduce bias in creatinine determination.

The continuous thresholds used here are specific to the European population and the Q values used in the EKFC equation. If these results were to be applied to other populations or if different estimating equations were used, the reference values would need to be re-estimated for that specific context. By deriving the 5th and 2.5th continuous age- and sex-adapted eGFR thresholds from eGFR reference values determined in a healthy population, the use of these thresholds will inherently identify at least 5% and 2.5% of individuals, respectively, as having abnormally low kidney function, despite being otherwise healthy. This is a limitation of using population-based statistical distributions to determine disease thresholds and, therefore, it is necessary to assess the associations of these thresholds with adverse clinical outcomes. Unfortunately, we do not have outcome data available and could not assess outcome measures in relation to the eGFR thresholds, such as the risk of kidney failure or mortality, which are required for a risk-based definition of CKD. Due to the absence of data on UACR which is required for diagnosis of CKD categories G1 to G2, our KDIGO eGFR threshold only pertains to the KDIGO CKD categories G3 to G5.

## CONCLUSION

Our results based on data from over 2.5 million individuals from a multinational European general population show that the application of age-adapted eGFR thresholds result in a markedly higher prevalence of reduced eGFR in younger adults and a lower prevalence in older adults when compared with the KDIGO eGFR threshold. Understanding the impact of age-adapted eGFR thresholds on both population health and for individual patient care can contribute to the discourse on the consideration of age in the definition of CKD. Importantly, further research is required to establish the association between age-adapted eGFR thresholds and adverse outcomes before considering their integration into the CKD definition.

## Supplementary Material

gfaf112_Supplemental_File

## Data Availability

The data used in this study are not available for sharing due to data protection laws and regulations.
