# Supplementary material for: Prevalence of reduced eGFR in European adults using KDIGO and age-adapted eGFR thresholds
Source: Nephrol Dial Transplant. 2025 Jul 17;40(12):2326–37. doi: 10.1093/ndt/gfaf112 (PMC12648058; doi:10.1093/ndt/gfaf112)

**Supplementary Materials**

**Supplemental Tables**

Table S1. Missingness of variables by study. Missingness is presented as a percentage.

**Table S2.** Estimated prevalence of reduced eGFR, calculated using the EKFC equation, presented per year of age by 5-year age intervals until the age of 85. Prevalence was estimated using four eGFR thresholds abbreviated as follows: KDIGO = KDIGO eGFR threshold, Categorical = categorical age-adapted eGFR threshold, 5^th^ Continuous = 5^th^ continuous age- and sex-adapted eGFR threshold, 2.5^th^ Continuous = 2.5^th^ continuous age- and sex-adapted eGFR threshold. All values are rounded to one decimal point. Results are presented as a percentage with 95% confidence intervals.

Table S3. Prevalence of reduced eGFR, calculated using the CKD-EPI_2009_ equation^A^, presented per year of age by 5-year age intervals, overall and by sex. Prevalence was estimated using four eGFR thresholds abbreviated as follows: KDIGO = KDIGO eGFR threshold, Categorical = categorical age-adapted eGFR threshold, 5^th^ Continuous = 5^th^ continuous age- and sex-adapted eGFR threshold, 2.5^th^ Continuous = 2.5^th^ continuous age- and sex-adapted eGFR threshold. All values are rounded to one decimal point. Results are presented as a percentage with 95% confidence intervals.

Table S4. Prevalence of reduced eGFR, calculated using the CKD-EPI_2021_ equation, presented per year of age by 5-year age intervals, overall and by sex. Prevalence was estimated using four eGFR thresholds abbreviated as follows: KDIGO = KDIGO eGFR threshold, Categorical = categorical age-adapted eGFR threshold, 5^th^ Continuous = 5^th^ continuous age- and sex-adapted eGFR threshold, 2.5^th^ Continuous = 2.5^th^ continuous age- and sex-adapted eGFR threshold. All values are rounded to one decimal point. Results are presented as a percentage with 95% confidence intervals.

**Supplemental Figures**

**Figure S1.** Prevalence of reduced eGFR, calculated using the EKFC equation, in those aged 18 to 96 years based on four thresholds to define reduced eGFR.

Figure S2. Prevalence of reduced eGFR, calculated using the EKFC equation, by study and sex, in those aged 18 to 96 years based on four eGFR thresholds.

Figure S3. Prevalence of reduced eGFR, calculated using the CKD-EPI_2009_ equation, overall and stratified by sex, in those aged 18 to 96 years based on four thresholds to define reduced eGFR.

Figure S4. Prevalence of reduced eGFR, calculated using the CKD-EPI_2021_ equation, overall and stratified by sex, in those aged 18 to 96 years based on four thresholds to define reduced eGFR.

**Supplementary tables**

Table S1. Missingness of variables by study. Missingness is presented as a percentage. Sex is categorized as men and women.

|  | Iceland CKD study | INCIPE | BIS | SHIP | Lifelines | The HUNT Study | The Tromsø Study | SCREAM | UK Biobank | Overall |
| --- | --- | --- | --- | --- | --- | --- | --- | --- | --- | --- |
| Age in years at moment of data collection | 0.0% | <1.0% | 0.0% | 0.0% | 1.1% | 0.0% | 0.0% | 0.0% | 0.0% | <1.0% |
| Sex | 0.0% | 0.0% | 0.0% | 0.0% | 1.1% | 0.0% | 0.0% | 0.0% | 0.0% | <1.0% |
| Serum creatinine | 0.0% | <1.0% | <1.0% | 0.16% | 4.1% | 1.2% | <1.0% | 0.0% | 6.4% | 1.5% |

INCIPE, Initiative on Nephropathy, of relevance to public health, which is Chronic, possibly in its Initial stages, and carries a Potential risk of major clinical Endpoints; BIS, The Berlin Initiative Study; SHIP, Study of Health in Pomeranzia; Lifelines, Lifelines Cohort Study; HUNT, Nord-Trøndelag Health Study; SCREAM, Stockholm CREAtinine Measurements project

**Table S2.** Estimated prevalence of reduced eGFR, calculated using the EKFC equation, presented per year of age by 5-year age intervals until the age of 85. Prevalence was estimated using four eGFR thresholds abbreviated as follows: KDIGO = KDIGO eGFR threshold, Categorical = categorical age-adapted eGFR threshold, 5^th^ Continuous = 5^th^ continuous age- and sex-adapted eGFR threshold, 2.5^th^ Continuous = 2.5^th^ continuous age- and sex-adapted eGFR threshold. All values are rounded to one decimal point. Results are presented as a percentage with 95% confidence intervals.

**Estimated prevalence of reduced eGFR using four eGFR thresholds, % (95% CI)**

| **Age**  **in years** |  | **KDIGO** | **Categorical** | **5^th^ Continuous** | **2.5^th^ Continuous** |
| --- | --- | --- | --- | --- | --- |
| **20** |  | 0.4 (0.3 - 0.4) | 3.2 (3.1 - 3.3) | 6.3 (6.2 - 6.5) | 3.8 (3.7 - 3.9) |
| **25** |  | 0.3 (0.3 - 0.3) | 2.4 (2.3 - 2.5) | 6.6 (6.5 - 6.7) | 3.9 (3.8 - 4) |
| **30** |  | 0.3 (0.3 - 0.3) | 2.5 (2.4 - 2.6) | 6.7 (6.7 - 6.8) | 4 (3.9 - 4) |
| **35** |  | 0.3 (0.3 - 0.4) | 2.6 (2.5 - 2.7) | 6.7 (6.6 - 6.8) | 3.9 (3.9 - 4) |
| **40** |  | 0.4 (0.4 - 0.4) | 0.3 (0.3 - 0.4) | 6.5 (6.4 - 6.6) | 3.9 (3.8 - 3.9) |
| **45** |  | 0.6 (0.6 - 0.6) | 0.6 (0.6 - 0.6) | 6.3 (6.2 - 6.4) | 3.8 (3.7 - 3.8) |
| **50** |  | 1 (1 - 1.1) | 1.1 (1 - 1.1) | 6.3 (6.3 - 6.4) | 3.8 (3.8 - 3.9) |
| **55** |  | 2 (1.9 - 2) | 2 (1.9 - 2) | 6.8 (6.7 - 6.9) | 4.2 (4.1 - 4.3) |
| **60** |  | 3.7 (3.7 - 3.8) | 3.7 (3.6 - 3.8) | 7.7 (7.7 - 7.8) | 5 (5 - 5.1) |
| **65** |  | 7.4 (7.3 - 7.5) | 7.6 (7.4 - 7.8) | 9.2 (9.2 - 9.3) | 6.3 (6.2 - 6.4) |
| **70** |  | 15 (14.8 - 15.1) | 2.9 (2.8 - 3) | 11.2 (11.1 - 11.3) | 7.9 (7.8 - 8) |
| **75** |  | 27.6 (27.3 - 27.8) | 6.9 (6.7 - 7.1) | 13.6 (13.5 - 13.8) | 9.9 (9.7 - 10) |
| **80** |  | 43.3 (43 - 43.6) | 14.2 (13.9 - 14.5) | 16.4 (16.2 - 16.6) | 12.1 (12 - 12.3) |
| **85** |  | 58.6 (58.2 - 59) | 24.2 (23.8 - 24.6) | 18.3 (18 - 18.6) | 13.4 (13.1 - 13.6) |

Table S3. Prevalence of reduced eGFR, calculated using the CKD-EPI_2009_ equation^A^, presented per year of age by 5-year age intervals, overall and by sex. Prevalence was estimated using four eGFR thresholds abbreviated as follows: KDIGO = KDIGO eGFR threshold, Categorical = categorical age-adapted eGFR threshold, 5^th^ Continuous = 5^th^ continuous age- and sex-adapted eGFR threshold, 2.5^th^ Continuous = 2.5^th^ continuous age- and sex-adapted eGFR threshold. All values are rounded to one decimal point. Results are presented as a percentage with 95% confidence intervals.

|  |  |  | **Overall** |  |  |
| --- | --- | --- | --- | --- | --- |
| **Age**  **in years** |  | **KDIGO** | **Categorical** | **5^th^ Continuous** | **2.5^th^ Continuous** |
| **20** |  | 0.2 (0.2 - 0.2) | 0.8 (0.8 - 0.9) | 6.5 (6.4 - 6.6) | 3.9 (3.8 - 4) |
| **25** |  | 0.2 (0.2 - 0.2) | 1.2 (1.2 - 1.3) | 6.5 (6.4 - 6.6) | 3.6 (3.5 - 3.6) |
| **30** |  | 0.3 (0.3 - 0.3) | 1.9 (1.8 - 1.9) | 6.6 (6.5 - 6.7) | 3.6 (3.5 - 3.7) |
| **35** |  | 0.4 (0.4 - 0.4) | 2.6 (2.5 - 2.7) | 6.5 (6.4 - 6.6) | 3.7 (3.6 - 3.8) |
| **40** |  | 0.5 (0.5 - 0.5) | 0.5 (0.4 - 0.5) | 6.1 (6 - 6.2) | 3.6 (3.5 - 3.6) |
| **45** |  | 0.8 (0.7 - 0.8) | 0.8 (0.7 - 0.8) | 5.8 (5.8 - 5.9) | 3.4 (3.3 - 3.4) |
| **50** |  | 1.2 (1.1 - 1.2) | 1.2 (1.1 - 1.2) | 6 (6 - 6.1) | 3.5 (3.4 - 3.5) |
| **55** |  | 1.8 (1.8 - 1.9) | 1.8 (1.8 - 1.9) | 6.7 (6.6 - 6.8) | 4 (3.9 - 4) |
| **60** |  | 3 (3 - 3.1) | 3 (2.9 - 3.1) | 7.6 (7.5 - 7.7) | 4.7 (4.7 - 4.8) |
| **65** |  | 5.5 (5.4 - 5.5) | 5.6 (5.5 - 5.8) | 8.9 (8.8 - 8.9) | 5.9 (5.8 - 5.9) |
| **70** |  | 10.4 (10.3 - 10.5) | 2.4 (2.4 - 2.5) | 10.8 (10.7 - 10.9) | 7.5 (7.4 - 7.6) |
| **75** |  | 19 (18.8 - 19.2) | 5.3 (5.2 - 5.4) | 13.3 (13.2 - 13.5) | 9.6 (9.4 - 9.7) |
| **80** |  | 30.6 (30.3 - 30.8) | 10.3 (10.1 - 10.6) | 15.9 (15.7 - 16.2) | 11.5 (11.3 - 11.7) |
| **85** |  | 42.9 (42.5 - 43.3) | 17.6 (17.3 - 18) | 17.8 (17.5 - 18.1) | 12.7 (12.4 - 12.9) |
|  |  |  | **Men** |  |  |
| **Age**  **(in years)** |  | **KDIGO** | **Categorical** | **5^th^ Continuous** | **2.5^th^ Continuous** |
| **20** |  | 0.2 (0.2 - 0.2) | 0.7 (0.7 - 0.8) | 4.7 (4.6 - 4.9) | 2.6 (2.5 - 2.7) |
| **25** |  | 0.3 (0.2 - 0.3) | 1.3 (1.2 - 1.4) | 5.8 (5.6 - 5.9) | 3 (2.9 - 3.1) |
| **30** |  | 0.3 (0.3 - 0.4) | 2 (1.9 - 2.1) | 6.2 (6.1 - 6.3) | 3.3 (3.2 - 3.4) |
| **35** |  | 0.4 (0.4 - 0.4) | 2.6 (2.5 - 2.7) | 5.9 (5.8 - 6) | 3.3 (3.3 - 3.4) |
| **40** |  | 0.6 (0.5 - 0.6) | 0.5 (0.5 - 0.6) | 5.6 (5.5 - 5.7) | 3.1 (3 - 3.2) |
| **45** |  | 0.8 (0.7 - 0.8) | 0.8 (0.7 - 0.8) | 5.5 (5.4 - 5.6) | 2.8 (2.8 - 2.9) |
| **50** |  | 1.1 (1 - 1.1) | 1.1 (1.1 - 1.2) | 5.5 (5.5 - 5.6) | 2.9 (2.9 - 3) |
| **55** |  | 1.7 (1.6 - 1.7) | 1.6 (1.6 - 1.7) | 6 (5.9 - 6.1) | 3.5 (3.4 - 3.5) |
| **60** |  | 2.8 (2.7 - 2.8) | 2.7 (2.6 - 2.8) | 7.1 (7 - 7.2) | 4.4 (4.3 - 4.5) |
| **65** |  | 5.1 (5 - 5.2) | 5.3 (5.1 - 5.6) | 8.8 (8.7 - 9) | 5.8 (5.7 - 5.9) |
| **70** |  | 9.8 (9.7 - 10) | 2.6 (2.5 - 2.7) | 11.1 (11 - 11.3) | 7.8 (7.7 - 8) |
| **75** |  | 18.2 (17.9 - 18.5) | 5.4 (5.2 - 5.5) | 14.1 (13.9 - 14.4) | 10.3 (10.1 - 10.6) |
| **80** |  | 29.8 (29.4 - 30.2) | 10.5 (10.2 - 10.8) | 17.6 (17.2 - 18) | 13 (12.7 - 13.3) |
| **85** |  | 42.4 (41.9 - 43) | 17.6 (17.1 - 18.1) | 20.4 (20 - 20.9) | 14.9 (14.5 - 15.3) |
|  |  |  | **Women** |  |  |
| **Age**  **(in years)** |  | **KDIGO** | **Categorical** | **5^th^ Continuous** | **2.5^th^ Continuous** |
| **20** |  | 0.2 (0.1 - 0.2) | 0.8 (0.8 - 0.9) | 7.8 (7.7 - 8) | 4.7 (4.6 - 4.9) |
| **25** |  | 0.2 (0.2 - 0.2) | 1.2 (1.2 - 1.3) | 7 (6.9 - 7.1) | 4 (3.9 - 4.1) |
| **30** |  | 0.2 (0.2 - 0.3) | 1.8 (1.7 - 1.8) | 7 (6.8 - 7.1) | 3.9 (3.8 - 3.9) |
| **35** |  | 0.3 (0.3 - 0.3) | 2.6 (2.5 - 2.7) | 7 (6.9 - 7.2) | 4 (3.9 - 4.1) |
| **40** |  | 0.5 (0.4 - 0.5) | 0.4 (0.4 - 0.5) | 6.6 (6.5 - 6.7) | 4 (3.9 - 4.1) |
| **45** |  | 0.7 (0.7 - 0.8) | 0.7 (0.7 - 0.8) | 6.2 (6.1 - 6.3) | 3.9 (3.8 - 4) |
| **50** |  | 1.2 (1.2 - 1.3) | 1.2 (1.2 - 1.3) | 6.5 (6.4 - 6.6) | 4 (3.9 - 4.1) |
| **55** |  | 2 (1.9 - 2) | 2 (1.9 - 2) | 7.3 (7.2 - 7.4) | 4.4 (4.4 - 4.5) |
| **60** |  | 3.3 (3.2 - 3.3) | 3.3 (3.2 - 3.4) | 8 (7.9 - 8.1) | 5 (4.9 - 5.1) |
| **65** |  | 5.8 (5.7 - 5.9) | 5.8 (5.6 - 6) | 8.9 (8.8 - 9) | 5.9 (5.8 - 6) |
| **70** |  | 10.9 (10.8 - 11.1) | 2.3 (2.2 - 2.4) | 10.5 (10.3 - 10.7) | 7.2 (7.1 - 7.4) |
| **75** |  | 19.6 (19.3 - 19.9) | 5.3 (5.1 - 5.5) | 12.7 (12.5 - 12.9) | 8.9 (8.7 - 9.1) |
| **80** |  | 31.1 (30.8 - 31.5) | 10.1 (9.9 - 10.4) | 14.7 (14.5 - 15) | 10.5 (10.2 - 10.7) |
| **85** |  | 43.2 (42.8 - 43.7) | 17.6 (17.2 - 18.1) | 16.1 (15.8 - 16.5) | 11.3 (11 - 11.6) |
|  |  |  |  |  |  |

^A^ Race was considered non-black in the calculation of eGFR using the CKD-EPI2009 equation as most studies had a predominantly White study population or did not/were not allowed to collect data on race.

Table S4. Prevalence of reduced eGFR, calculated using the CKD-EPI_2021_ equation, presented per year of age by 5-year age intervals, overall and by sex. Prevalence was estimated using four eGFR thresholds abbreviated as follows: KDIGO = KDIGO eGFR threshold, Categorical = categorical age-adapted eGFR threshold, 5^th^ Continuous = 5^th^ continuous age- and sex-adapted eGFR threshold, 2.5^th^ Continuous = 2.5^th^ continuous age- and sex-adapted eGFR threshold. All values are rounded to one decimal point. Results are presented as a percentage with 95% confidence intervals.

|  |  |  | **Overall** |  |  |
| --- | --- | --- | --- | --- | --- |
| **Age**  **in years** |  | **KDIGO** | **Categorical** | **5^th^ Continuous** | **2.5^th^ Continuous** |
| **20** |  | 0.2 ( 0.1 - 0.2 ) | 0.6 ( 0.6 - 0.7 ) | 7.2 ( 7.1 - 7.3 ) | 4.6 ( 4.5 - 4.7 ) |
| **25** |  | 0.2 ( 0.2 - 0.2 ) | 0.8 ( 0.8 - 0.9 ) | 6.9 ( 6.8 - 7 ) | 4.4 ( 4.3 - 4.5 ) |
| **30** |  | 0.2 ( 0.2 - 0.2 ) | 1.2 ( 1.1 - 1.2 ) | 6.9 ( 6.9 - 7 ) | 4.5 ( 4.4 - 4.6 ) |
| **35** |  | 0.3 ( 0.3 - 0.3 ) | 1.7 ( 1.7 - 1.8 ) | 7.1 ( 7 - 7.2 ) | 4.6 ( 4.5 - 4.7 ) |
| **40** |  | 0.4 ( 0.4 - 0.4 ) | 0.3 ( 0.3 - 0.4 ) | 6.8 ( 6.8 - 6.9 ) | 4.3 ( 4.2 - 4.4 ) |
| **45** |  | 0.5 ( 0.5 - 0.5 ) | 0.5 ( 0.5 - 0.5 ) | 6.4 ( 6.4 - 6.5 ) | 4 ( 3.9 - 4.1 ) |
| **50** |  | 0.7 ( 0.7 - 0.8 ) | 0.7 ( 0.7 - 0.8 ) | 6.4 ( 6.3 - 6.5 ) | 4.1 ( 4.1 - 4.2 ) |
| **55** |  | 1.1 ( 1.1 - 1.2 ) | 1.2 ( 1.1 - 1.2 ) | 6.9 ( 6.8 - 7 ) | 4.7 ( 4.6 - 4.8 ) |
| **60** |  | 1.9 ( 1.8 - 1.9 ) | 1.8 ( 1.8 - 1.9 ) | 7.9 ( 7.8 - 8 ) | 5.5 ( 5.4 - 5.6 ) |
| **65** |  | 3.5 ( 3.4 - 3.6 ) | 3.6 ( 3.5 - 3.7 ) | 9.5 ( 9.4 - 9.6 ) | 6.6 ( 6.6 - 6.7 ) |
| **70** |  | 6.9 ( 6.8 - 7 ) | 1.8 ( 1.8 - 1.9 ) | 11.7 ( 11.6 - 11.8 ) | 8.3 ( 8.2 - 8.5 ) |
| **75** |  | 13.2 ( 13 - 13.4 ) | 3.9 ( 3.7 - 4 ) | 14.3 ( 14.1 - 14.5 ) | 10.5 ( 10.4 - 10.7 ) |
| **80** |  | 22.7 ( 22.4 - 22.9 ) | 7.5 ( 7.3 - 7.7 ) | 16.8 ( 16.6 - 17.1 ) | 12.6 ( 12.4 - 12.8 ) |
| **85** |  | 33.8 ( 33.5 - 34.2 ) | 13.1 ( 12.8 - 13.4 ) | 18.7 ( 18.5 - 19 ) | 13.7 ( 13.5 - 13.9 ) |
|  |  |  | **Men** |  |  |
| **Age**  **(in years)** |  | **KDIGO** | **Categorical** | **5^th^ Continuous** | **2.5^th^ Continuous** |
| **20** |  | 0.2 ( 0.2 - 0.2 ) | 0.6 ( 0.6 - 0.7 ) | 5.6 ( 5.5 - 5.8 ) | 3.7 ( 3.5 - 3.8 ) |
| **25** |  | 0.2 ( 0.2 - 0.3 ) | 0.9 ( 0.9 - 1 ) | 6.4 ( 6.3 - 6.5 ) | 4.2 ( 4.1 - 4.3 ) |
| **30** |  | 0.3 ( 0.3 - 0.3 ) | 1.3 ( 1.3 - 1.4 ) | 6.8 ( 6.7 - 6.9 ) | 4.7 ( 4.6 - 4.8 ) |
| **35** |  | 0.3 ( 0.3 - 0.4 ) | 1.9 ( 1.8 - 2 ) | 6.7 ( 6.5 - 6.8 ) | 4.7 ( 4.6 - 4.8 ) |
| **40** |  | 0.4 ( 0.4 - 0.5 ) | 0.4 ( 0.3 - 0.4 ) | 6.3 ( 6.1 - 6.4 ) | 4.2 ( 4.1 - 4.2 ) |
| **45** |  | 0.6 ( 0.5 - 0.6 ) | 0.6 ( 0.5 - 0.6 ) | 6 ( 5.9 - 6.1 ) | 3.6 ( 3.6 - 3.7 ) |
| **50** |  | 0.8 ( 0.7 - 0.8 ) | 0.8 ( 0.7 - 0.8 ) | 6.1 ( 6 - 6.2 ) | 3.7 ( 3.6 - 3.7 ) |
| **55** |  | 1.1 ( 1.1 - 1.2 ) | 1.1 ( 1.1 - 1.2 ) | 6.6 ( 6.4 - 6.7 ) | 4.3 ( 4.2 - 4.4 ) |
| **60** |  | 1.9 ( 1.8 - 1.9 ) | 1.8 ( 1.7 - 1.9 ) | 7.6 ( 7.4 - 7.7 ) | 5.4 ( 5.3 - 5.4 ) |
| **65** |  | 3.5 ( 3.4 - 3.6 ) | 3.7 ( 3.5 - 3.8 ) | 9.4 ( 9.3 - 9.5 ) | 6.7 ( 6.6 - 6.8 ) |
| **70** |  | 6.9 ( 6.8 - 7.1 ) | 2 ( 1.9 - 2.1 ) | 12.1 ( 11.9 - 12.3 ) | 8.7 ( 8.5 - 8.8 ) |
| **75** |  | 13.2 ( 13 - 13.5 ) | 4.1 ( 3.9 - 4.3 ) | 15.2 ( 14.9 - 15.4 ) | 11.3 ( 11.1 - 11.6 ) |
| **80** |  | 22.6 ( 22.2 - 22.9 ) | 8 ( 7.7 - 8.3 ) | 18.2 ( 17.9 - 18.6 ) | 14.1 ( 13.8 - 14.4 ) |
| **85** |  | 33.9 ( 33.4 - 34.5 ) | 13.6 ( 13.2 - 14.1 ) | 21 ( 20.5 - 21.4 ) | 15.8 ( 15.4 - 16.2 ) |
|  |  |  | **Women** |  |  |
| **Age**  **(in years)** |  | **KDIGO** | **Categorical** | **5^th^ Continuous** | **2.5^th^ Continuous** |
| **20** |  | 0.1 ( 0.1 - 0.2 ) | 0.6 ( 0.6 - 0.7 ) | 8.3 ( 8.1 - 8.4 ) | 5.3 ( 5.2 - 5.4 ) |
| **25** |  | 0.2 ( 0.1 - 0.2 ) | 0.7 ( 0.7 - 0.8 ) | 7.2 ( 7.1 - 7.3 ) | 4.6 ( 4.5 - 4.7 ) |
| **30** |  | 0.2 ( 0.2 - 0.2 ) | 1 ( 0.9 - 1.1 ) | 7.1 ( 7 - 7.2 ) | 4.4 ( 4.3 - 4.5 ) |
| **35** |  | 0.2 ( 0.2 - 0.2 ) | 1.6 ( 1.5 - 1.7 ) | 7.4 ( 7.3 - 7.5 ) | 4.5 ( 4.4 - 4.6 ) |
| **40** |  | 0.3 ( 0.3 - 0.3 ) | 0.3 ( 0.3 - 0.3 ) | 7.3 ( 7.2 - 7.4 ) | 4.4 ( 4.3 - 4.5 ) |
| **45** |  | 0.5 ( 0.4 - 0.5 ) | 0.4 ( 0.4 - 0.5 ) | 6.8 ( 6.7 - 6.9 ) | 4.3 ( 4.3 - 4.4 ) |
| **50** |  | 0.7 ( 0.7 - 0.8 ) | 0.7 ( 0.7 - 0.8 ) | 6.7 ( 6.6 - 6.8 ) | 4.6 ( 4.5 - 4.6 ) |
| **55** |  | 1.1 ( 1.1 - 1.2 ) | 1.2 ( 1.1 - 1.2 ) | 7.2 ( 7.1 - 7.3 ) | 5 ( 5 - 5.1 ) |
| **60** |  | 1.9 ( 1.9 - 2 ) | 1.9 ( 1.8 - 2 ) | 8.3 ( 8.2 - 8.4 ) | 5.6 ( 5.6 - 5.7 ) |
| **65** |  | 3.5 ( 3.4 - 3.6 ) | 3.5 ( 3.3 - 3.6 ) | 9.6 ( 9.5 - 9.7 ) | 6.5 ( 6.4 - 6.6 ) |
| **70** |  | 6.9 ( 6.7 - 7 ) | 1.6 ( 1.6 - 1.7 ) | 11.3 ( 11.2 - 11.5 ) | 8 ( 7.9 - 8.2 ) |
| **75** |  | 13.2 ( 12.9 - 13.4 ) | 3.7 ( 3.5 - 3.8 ) | 13.5 ( 13.3 - 13.7 ) | 9.9 ( 9.7 - 10.1 ) |
| **80** |  | 22.7 ( 22.4 - 23.1 ) | 7.1 ( 6.9 - 7.4 ) | 15.8 ( 15.5 - 16.1 ) | 11.5 ( 11.3 - 11.8 ) |
| **85** |  | 33.8 ( 33.3 - 34.2 ) | 12.8 ( 12.4 - 13.1 ) | 17.3 ( 17 - 17.6 ) | 12.4 ( 12.1 - 12.7 ) |
|  |  |  |  |  |  |

**Figure S1.** Prevalence of reduced eGFR, calculated using the EKFC equation, in those aged 18 to 96 years based on four thresholds to define reduced eGFR: the KDIGO eGFR threshold (eGFR < 60 mL/min/1.73 m2), the categorical age-adapted eGFR threshold (eGFR < 75 mL/min/1.73 m2 for those aged below 40 years, eGFR < 60 mL/min/1.73 m2 for those aged 40 to 65 years, eGFR < 45 mL/min/1.73 m2 for those aged above 65 years), the 5th continuous age- and sex-adapted eGFR threshold (5th percentile of eGFR from a healthy population) and the 2.5th continuous age- and sex-adapted eGFR threshold (2.5th percentile of eGFR from a healthy population). Because the KDIGO and categorical age-adapted definition have the same eGFR threshold from the ages of 40 to 65 years, the lines for this age range are overlapping as is illustrated by the dashed purple line overlapping the solid green line.





Figure S2. Prevalence of reduced eGFR, calculated using the EKFC equation, by study and sex in those aged 18 to 96 years based on four eGFR thresholds. Prevalence is estimated using the KDIGO eGFR threshold, the categorical age-adapted eGFR threshold, and the continuous age- and sex-adapted eGFR threshold. Because the KDIGO and categorical age-adapted definition have the same eGFR threshold from the ages of 40 to 65 years, the lines for this age range are overlapping as is illustrated by the dashed purple line overlapping the solid green line. Figures are presented in alphabetical order by study name: A. the BIS; B. the Hunt study; C. the Iceland CKD study cohort; D. the INCIPE study; E. the Lifelines cohort; F. SCREAM; G. SHIP; H. the Tromsø study; I. the UK Biobank.

1. The BIS





1. The HUNT Study





1. The Iceland CKD Study





1. The INCIPE Study





1. The Lifelines cohort^A^


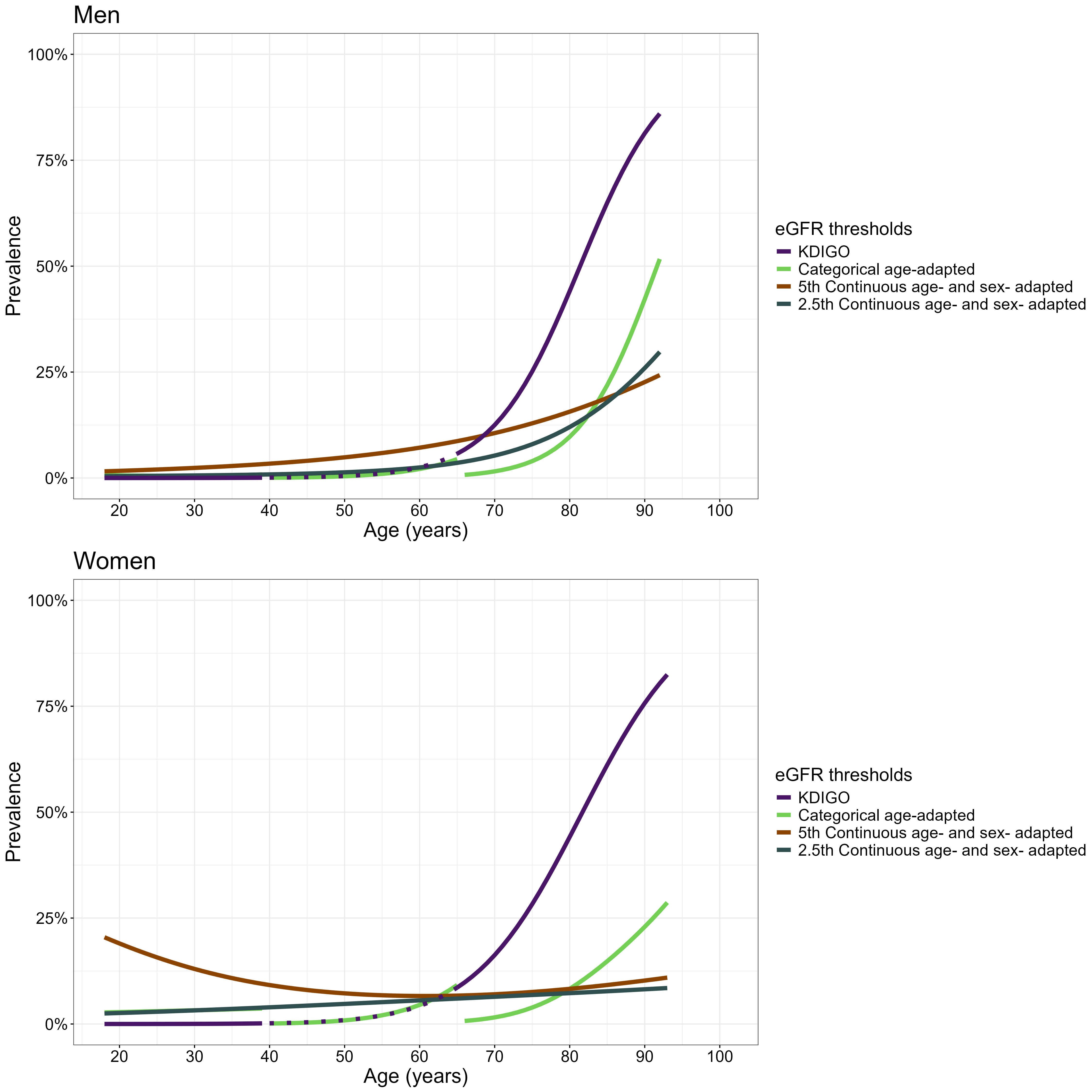


^A^ Serum creatinine was only available to one decimal point and as a result the estimation of GFR was less precise. When using the continuous age- and sex-adapted eGFR thresholds, prevalence estimates were inconsistent due to the limited eGFR values calculated when using serum creatinine to one decimal point so the Lifelines cohort data was modelled with less flexibility than other studies to avoid an over-fitted line.

1. SCREAM





1. SHIP





1. The Tromsø Study





1. The UK Biobank





Figure S3. Prevalence of reduced eGFR, calculated using the CKD-EPI_2009_ equation, overall and stratified by sex, in those aged 18 to 96 years based on four thresholds to define reduced eGFR: the KDIGO eGFR threshold (eGFR < 60 mL/min/1.73 m2), the categorical age-adapted eGFR threshold (eGFR < 75 mL/min/1.73 m2 for those aged below 40 years, eGFR < 60 mL/min/1.73 m2 for those aged 40 to 65 years, eGFR < 45 mL/min/1.73 m2 for those aged above 65 years), the 5th continuous age- and sex-adapted eGFR threshold (5th percentile of eGFR from a healthy population) and the 2.5th continuous age- and sex-adapted eGFR threshold (2.5th percentile of eGFR from a healthy population). Because the KDIGO and categorical age-adapted definition have the same eGFR threshold from the ages of 40 to 65 years, the lines for this age range are overlapping as is illustrated by the dashed purple line overlapping the solid green line.







Figure S4. Prevalence of reduced eGFR, calculated using the CKD-EPI_2021_ equation, overall and stratified by sex, in those aged 18 to 96 years based on four thresholds to define reduced eGFR: the KDIGO eGFR threshold (eGFR < 60 mL/min/1.73 m2), the categorical age-adapted eGFR threshold (eGFR < 75 mL/min/1.73 m2 for those aged below 40 years, eGFR < 60 mL/min/1.73 m2 for those aged 40 to 65 years, eGFR < 45 mL/min/1.73 m2 for those aged above 65 years), the 5th continuous age- and sex-adapted eGFR threshold (5th percentile of eGFR from a healthy population) and the 2.5th continuous age- and sex-adapted eGFR threshold (2.5th percentile of eGFR from a healthy population). Because the KDIGO and categorical age-adapted definition have the same eGFR threshold from the ages of 40 to 65 years, the lines for this age range are overlapping as is illustrated by the dashed purple line overlapping the solid green line.


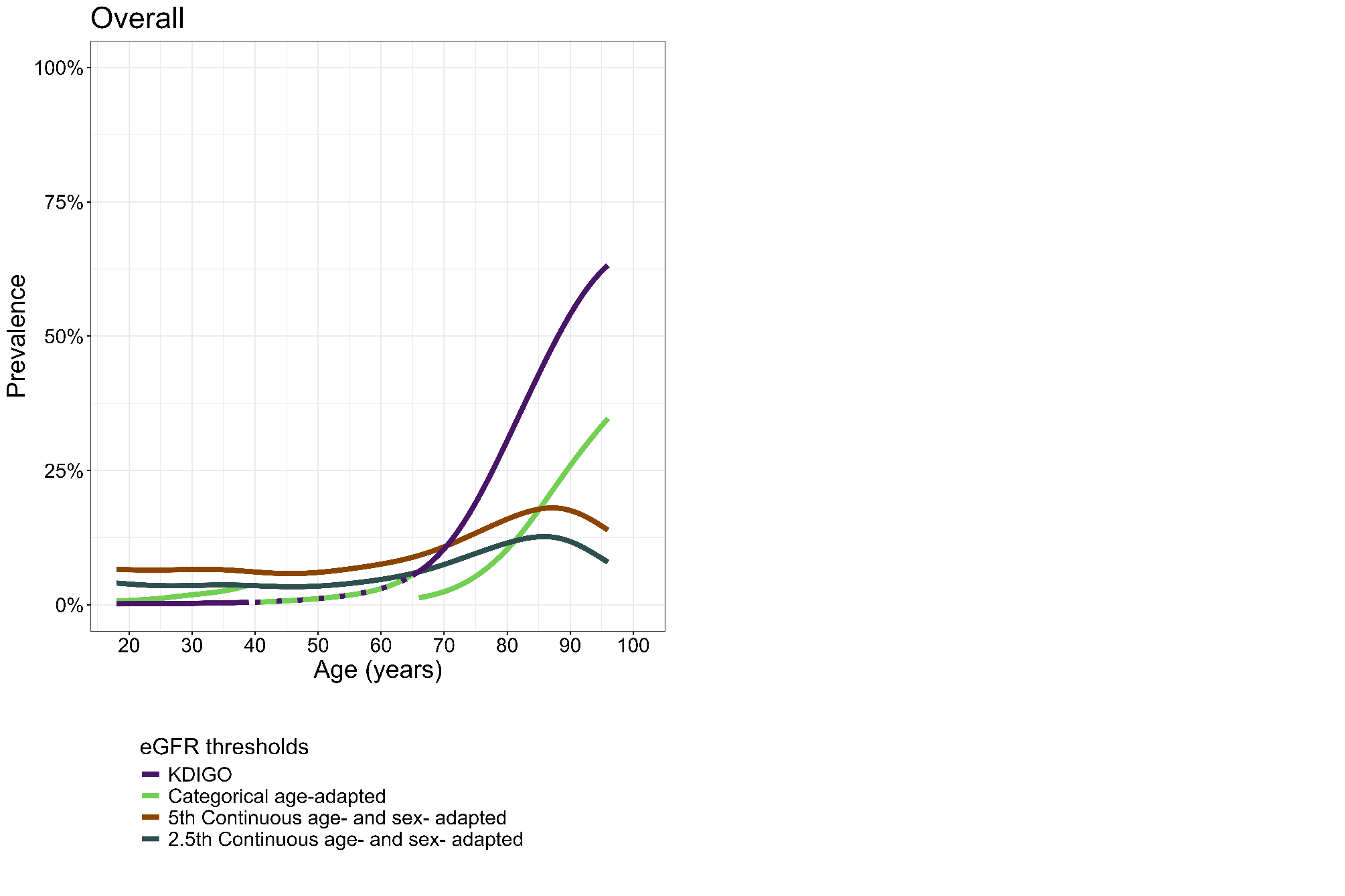

Supplement: gfaf112_Supplemental_File [file gfaf112_supplemental_file.docx]
